# Identification of conserved, centrosome-targeting ASH domains in TRAPPII complex subunits and TRAPPC8

**DOI:** 10.1186/2046-2530-3-6

**Published:** 2014-06-18

**Authors:** Kenneth B Schou, Stine K Morthorst, Søren T Christensen, Lotte B Pedersen

**Affiliations:** 1 Department of Biology, University of Copenhagen, Universitetsparken 13, Copenhagen, Denmark; 2 Center for Experimental Bioinformatics, Department of Biochemistry and Molecular Biology, University of Southern Denmark, Odense, Denmark

**Keywords:** TRAPPII complex, ASH domain, Vesicle trafficking, Cilia, Rab8, TPR repeat, Golgi, Centrosome, Rabin8, MSP domain

## Abstract

**Background:**

Assembly of primary cilia relies on vesicular trafficking towards the cilium base and intraflagellar transport (IFT) between the base and distal tip of the cilium. Recent studies have identified several key regulators of these processes, including Rab GTPases such as Rab8 and Rab11, the Rab8 guanine nucleotide exchange factor Rabin8, and the transport protein particle (TRAPP) components TRAPPC3, -C9, and -C10, which physically interact with each other and function together with Bardet Biedl syndrome (BBS) proteins in ciliary membrane biogenesis. However, despite recent advances, the exact molecular mechanisms by which these proteins interact and target to the basal body to promote ciliogenesis are not fully understood.

**Results:**

We surveyed the human proteome for novel ASPM, SPD-2, Hydin (ASH) domain-containing proteins. We identified the TRAPP complex subunits TRAPPC8, -9, -10, -11, and -13 as novel ASH domain-containing proteins. In addition to a C-terminal ASH domain region, we predict that the N-terminus of TRAPPC8, -9, -10, and -11, as well as their yeast counterparts, consists of an α-solenoid bearing stretches of multiple tetratricopeptide (TPR) repeats. Immunofluorescence microscopy analysis of cultured mammalian cells revealed that exogenously expressed ASH domains, as well as endogenous TRAPPC8, localize to the centrosome/basal body. Further, depletion of TRAPPC8 impaired ciliogenesis and GFP-Rabin8 centrosome targeting.

**Conclusions:**

Our results suggest that ASH domains confer targeting to the centrosome and cilia, and that TRAPPC8 has cilia-related functions. Further, we propose that the yeast TRAPPII complex and its mammalian counterpart are evolutionarily related to the bacterial periplasmic trafficking chaperone PapD of the usher pili assembly machinery.

## Background

The primary cilium is a microtubule-based sensory organelle that extends from the mother centriole, projecting from the cell surface into the extracellular environment [1]. The assembly of primary cilia is a multistep process initiated by docking of the centriole distal end to a vesicle or membrane patch that subsequently expands and surrounds the axoneme as it elongates by intraflagellar transport (IFT) [2,3]. In many cell types the proximal part of the mature cilium resides in the cytoplasm within an invagination of the plasma membrane known as the ciliary pocket. This region is a unique site for vesicular trafficking by endo- and exocytosis that plays a critical role in ciliary membrane homeostasis and function [4,5].

Primary cilia have become the focus of mounting studies owing to their role in coordinating cellular signaling processes during development and tissue homeostasis, and consequently, their association with a constellation of genetic diseases and syndromes called ciliopathies [6,7]. These include the Bardet-Biedl syndrome (BBS), characterized by obesity, renal anomalies, cognitive defects, post-axial polydactyly, hypogonadism, retinal degeneration, and anosmia [8]. Seven BBS gene products (BBS1, BBS2, BBS4, BBS5, BBS7, BBS8, and BBS9) and BBIP10 reside in a stable complex called the BBSome [9,10]. The BBSome interacts with the Rab8 guanine nucleotide exchange factor Rabin8, as well as several other proteins, and mediates trafficking of membrane proteins to and from cilia [9,11,12]. Although numerous proteins have been implicated in vesicular transport to cilia (reviewed in [13]), Rab8 seems to be a key player in the very first stages of ciliary membrane biogenesis. Rab8 is targeted to the primary cilium during early ciliogenesis followed by a gradual loss from the cilium as the organelle matures [14]. Rab8 is activated by Rabin8 [15], which is recruited to the centrosome and activated by a mechanism involving Rab11 and homologs of the yeast transport protein particle II (TRAPPII) complex subunits [14,16].

Current evidence indicates that the yeast TRAPPII complex consists of a core containing the seven TRAPPI subunits (Trs20, Trs23, Trs31, Trs33, Bet3a, Bet3b, Bet5) as well as the four TRAPPII-specific subunits Trs65, Trs120, Trs130, and Tca17 [17,18]. Functional and biochemical studies have indicated that yeast TRAPPI and TRAPPII complexes are distinct vesicle tethering entities that function in ER-Golgi and intra-Golgi plus endosome-Golgi transport, respectively [17]. Yeast also contains a third TRAPP complex, TRAPPIII, which consists of TRAPPI and Trs85 and functions in autophagy [17]. The composition and function of mammalian TRAPP complexes is less clear, and there is some uncertainty as to how many distinct TRAPP complexes exist in mammalian cells [17]. It is clear, however, that mammalian homologs of the yeast TRAPPI subunits exist in one or more large complexes that also include several homologs of yeast TRAPPII-specific subunits, including Trs120 (TRAPPC9), Trs130 (TRAPPC10), Tca17 (TRAPPC2L) in addition to the Trs130-related TRAPPC11 (C4orf41) and the Trs65-related TRAPPC13 (C5orf44) [17-19]. Furthermore, although Trs85 seems to be absent from the yeast TRAPPII complex, an ortholog of Trs85 (TRAPPC8) appears to be part of the mammalian TRAPP complex since TRAPPC8 was reported to interact with TRAPPC2 (Trs20 homolog) [20] as well as with TRAPPC2L (Tca17 homolog) and TRAPPC13 [18]. Thus in mammalian cells, TRAPPC8 engages in a complex with both TRAPPI and TRAPPII components. Curiously, TRAPPC8 does not appear to interact with the TRAPPII component TRAPPC9 [14,20], suggesting the existence of distinct TRAPPII-like complexes in mammalian cells. Mammalian TRAPPC8 differs from yeast Trs85 in that it contains a C-terminal extension related to the C-terminus of TRAPPC9, which may explain its association with TRAPPII-specific subunits [18].

Similar to yeast TRAPP complexes, mammalian TRAPP has been implicated in various vesicle trafficking pathways [17]. For example, it was shown that depletion of TRAPPC3, TRAPPC9, or TRAPPC10 in cultured cells impaired primary ciliary membrane biogenesis by inhibiting Rabin8 recruitment to the centrosome [14]. While the possible roles of other TRAPP components in ciliogenesis are unclear, several additional proteins involved in ciliary membrane biogenesis interact with Rab8/Rabin8 (reviewed in [13]). These include the centriole distal appendage protein Cep164 [21] and components of the exocyst complex [22] as well as oculocerebrorenal syndrome of Lowe protein, OCRL1, mutations in which cause a rare X-linked disorder characterized by mental retardation, renal tubulopathy, and congenital cataracts [23]. OCRL1 resides in complexes with various Rab proteins [24,25], with preference for Rab8a, via a binding interface consisting of one α-helix and an adjacent ASH (ASPM, SPD-2, Hydin) domain [26], a novel family of remote homologs of the immunoglobulin (Ig)-like seven-stranded beta sandwich fold superfamily of the nematode major sperm proteins (MSPs) [27,28]. Although poorly defined at present, the MSP fold is believed to form a protein-protein interaction interface involved in cellular signaling and trafficking activities [28,29]. A previous computational survey identified 13 human ASH-containing proteins [27], all of which are confined to three subcellular compartments, namely the centrosome, Golgi, and the cilium, suggesting that the ASH domain is involved in cilia-related functions [27]. Indeed, OCRL1 was recently shown to be required for ciliogenesis and to promote protein trafficking to the primary cilium via a Rab8 and IPIP27/Ses-dependent mechanism [30]. Another ASH-domain-containing protein, the hydrocephalus-associated protein, Hydin, is required for formation of central pair projections of motile cilia and, in turn, for regulating ciliary motility [31-33], whereas the ASH domain-containing ASPM protein is a centrosomal and microtubule-associated protein that causes microcephaly when defective or absent [34].

We here revisit the ciliary family of ASH domain-containing proteins in humans and yeast and unveil the existence of ASH domains in several members of the TRAPP complex family of proteins. Our database searches identify conserved ASH domains in the C-terminus of mammalian TRAPPC8, -9, -10, -11, and -13, as well as in the yeast TRAPPII complex proteins Trs65, Trs120, and Trs130, none of which were previously assigned any protein structure. We find that the ASH domain in most of these proteins is preceeded by an N-terminal region containing long α-helical matrices exhibiting high levels of helicity and TPR repeat propensity. We further provide evidence that the ASH domains of TRAPPC10 and TRAPPC11 localize to the centrosome when expressed as fusion proteins in mammalian cells and that their expression leads to defects in microtubule organization. Finally, we find that endogenous TRAPPC8 localizes to the centrosome/basal body in cultured cells, and that TRAPPC8 depletion impairs ciliogenesis and targeting of GFP-Rabin8 to the centrosome. Our results corroborate the notion that the ASH domain is involved in microtubule- and cilia-related processes and provide insight into the mechanism by which mammalian TRAPPII homologs target to the centrosome/basal body. Our results further suggest that the yeast TRAPPII complex and its mammalian counterpart are evolutionarily related to the bacterial periplasmic trafficking chaperone PapD of the usher pili assembly machinery [35].

## Methods

### Bioinformatics

Profile-to-profile hidden Markov model (HMM)-HMM searches against the PFAMA database (http://pfam.sanger.ac.uk) were performed using HHpred [36,37] with default settings. Multiple sequence alignments (MSA) were generated using MAFFT [38,39], edited in Jalview [40], and the consensus of the alignment calculated and colored using ClustalX, as implemented in Jalview. Secondary structure information and structural alignment were predicted using HHpred [37]. For homology modeling of three-dimensional (3D) structures, Modeller [41,42] was employed and templates were chosen based on highest probability and significantly low E value. Discovery Studio 3.5 Visualizer was used for analysis of resulting 3D model coordinates.

### PCR and cloning procedures

For generation of plasmids coding for Myc-tagged ASH domains from human TRAPPC10 (amino acid residues 1000-1259) and TRAPPC11 (residues 701-1133; transcript variant 1), the corresponding cDNA regions were PCR-amplified from retinal pigment epithelial cell cDNA [43] by standard procedures using forward (CA*GAATTC*TCCCCATCTACAGCAAGCAGTC for TRAPPC10; CA*GAATTC*TCTTAAATTGGCAGGGAGGAGGAGGA for TRAPPC11) and reverse (CA*GGTACC*TCATGTTACACTGACTTCCAGG for TRAPPC10; CA*GGTACC*TCATGCAGCAGCAATAGAGGTAT for TRAPPC11) primers containing *Eco*R1 and *Kpn*1 restriction sites (italics), respectively. The PCR products were cloned into pCMV-Myc (Clontech laboratories, Inc.) and transformed into *Escherichia coli* DH10α using standard procedures. Plasmids from recombinant bacteria were purified using endotoxin-free plasmid DNA purification kit (NucleoBond Xtra Midi EF) from Macherey-Nagel and the inserts sequenced at Eurofins MWG Operon.

### Mammalian cell culture

The retinal pigment epithelial (RPE) cells used (lab stock) were derived from the immortalized hTERT RPE-1 cell-line and cultured as described previously [43].

### Immunofluorescence microscopy

For immunofluorescence microscopy analysis of cells expressing ASH domain fusion proteins RPE cells were seeded on coverslips, transfected with plasmids encoding Myc-TRAPPC10-ASH or Myc-TRAPPC11-ASH (see above) and serum starved for 24 h. Cells were fixed with methanol or 4% PFA and subjected to immunofluorescence microscopy as described [43] using rabbit monoclonal antibody specific for Myc (1:500 dilution; Cell Signaling) and mouse monoclonal antibodies specific for α-tubulin (1:4,000 dilution; Sigma), acetylated-tubulin (1:4,000 dilution; Sigma) or p150^Glued^ (1:250 dilution; BD Biosciences). To study the localization of endogenous TRAPPC8 RPE cells were seeded on coverslips and incubated in serum-depleted medium for 24 h to induce cilia formation. Cells were fixed with methanol and subjected to immunofluorescence microscopy as described [43] using rabbit polyclonal antibody specific for TRAPPC8 (1:100 dilution; Sigma), rat monoclonal antibody specific for EB3 (1:300 dilution; Absea clone KT36), and mouse monoclonal antibodies specific for acetylated-tubulin (1:5,000 dilution; Sigma) and p150^Glued^ (1:500 dilution; BD Biosciences). Imaging was performed with a motorized Olympus BX63 upright microscope equipped with a DP72 color, 12.8 megapixel, 4140 × 3096 resolution camera and differential interference contrast (DIC). The software used was Olympus CellSens dimension. Images were processed for publication using Adobe Photoshop CS4 version 11.0.

### TRAPPC8 knock-down, GFP-Rabin8 expression, SDS-PAGE, and western blot

For TRAPPC8 knock-down experiments, RPE cells were seeded and subjected to transfection with 100 nM esiRNA specifically targeting TRAPP8C (Cat# EHU065741; Sigma) or control siRNA (5′-UAA UGU AUU GGA ACG CAU ATT-3′; Eurofins MWG Operon) using DharmaFECT Duo Transfection Reagent (Thermo Scientific) essentially as described [43]. Cells were then incubated in serum-depleted medium for 24 h and either PFA-fixed and analyzed by immunofluorescence microscopy with acetylated tubulin antibody as described above or lysed using 0.5% SDS; the lysates were subsequently analyzed by SDS-PAGE and western blotting as described previously [44]. Rabbit polyclonal antibody specific for TRAPPC8 (1:500 dilution; Sigma) and mouse monoclonal antibody specific for α-tubulin (1:2,000 dilution; Sigma) were used for western blot. Blots were scanned and processed for publication using Adobe Photoshop CS6 version 13.0 and Adobe Illustrator CS6 version 16.0.0. For experiments with GFP-Rabin8 plasmid RPE cells were seeded and transfected with 100 nM esiRNA or control siRNA as described above. After 30 h, cells were transfected with plasmid encoding GFP-Rabin8 [14] for another 16 h. Prior to fixation with PFA cells were serum starved for 1 h and subjected to immunofluorescence microscopy with p150^Glued^ antibody as described above, and the number of GFP-positive centrosomes in GFP-Rabin8 expressing cells was scored.

## Results

### Identification of ASH domains in the C-terminus of known and putative TRAPPII components

Sequence homologies between the ASH domain and yeast TRAPPII-specific components and their mammalian counterparts were readily attained by searching the PFAM database of protein families [45] using the human DLEC1 amino acid sequence as a search query in the HHpred server (http://toolkit.tuebingen.mpg.de/hhpred) (Additional file 1: Figure S1). We chose DLEC1 as search query because this protein was identified with high confidence as an ASH domain-containing protein in a bioinformatics analysis [27]. However, the molecular function of DLEC1 is largely unknown. Specifically, a HMM based profile-to-profile search with a minimal portion of human DLEC1 (amino acids 769-980; [27]) carrying the second ASH module produced high probability sequence similarities to the TRAPPC9-Trs120 family of the PFAM entry PF08626 (local search algorithm: probability 97.47, E = 0.035), as well as to a profile of bacterial PapD/FimC (PF14874) and its eukaryotic derivative, the MSP domain (PF00635) (both having E < 1 × 10^-5^) [28]. Since we found that the ASH and MSP domains resemble the same conserved domain family, we have chosen to use the terms ASH and MSP interchangeably. An analogous search using the global search mode recovered essentially the same matches yielding higher score parameters to TRAPPC9-Trs120 (probability 98.10, E = 3.6 × 10^-5^). This indicates that the family of TRAPPII proteins, encompassing metazoan TRAPPC9 homologs and yeast Trs120, bear homology by sequence to the ASH domain. Indeed, a reciprocal profile-to-profile search seeded with, for example, yeast Trs120 (amino acids 660-948) identified, besides TRAPPC9-Trs120, the PapD family as the highest scoring hit (local/global search modes: probability 97.78/97.22, E = 8.6 × 10^-4^/4.4 × 10^-4^). Likewise, using a longer stretch of the Trs120 C-terminus (amino acids 486-1166) as a search query, employing three MSA generation iterations, we identified a portion of human Hydin (amino acids 361-892) containing three ASH domains (global search probability 96.50, E = 0.017), supporting the existence of the ASH domain in Trs120/TRAPPC9. Interestingly, this search also identified remote sequence similarities to additional or putative TRAPPII subunits, including human TRAPPC13/C5orf44 (local probability 99.41, E = 9.2 × 10^-12^), C4orf44/Gryzun (TRAPPC11) (local probability 99.39, E = 1.1 × 10^-9^) as well as TRAPPC8 (local probability 100, E = 7.8 × 10^-32^). These findings raise the possibility that mammalian TRAPP possesses multiple paralogous ASH domain-bearing components.

**Figure 1:**
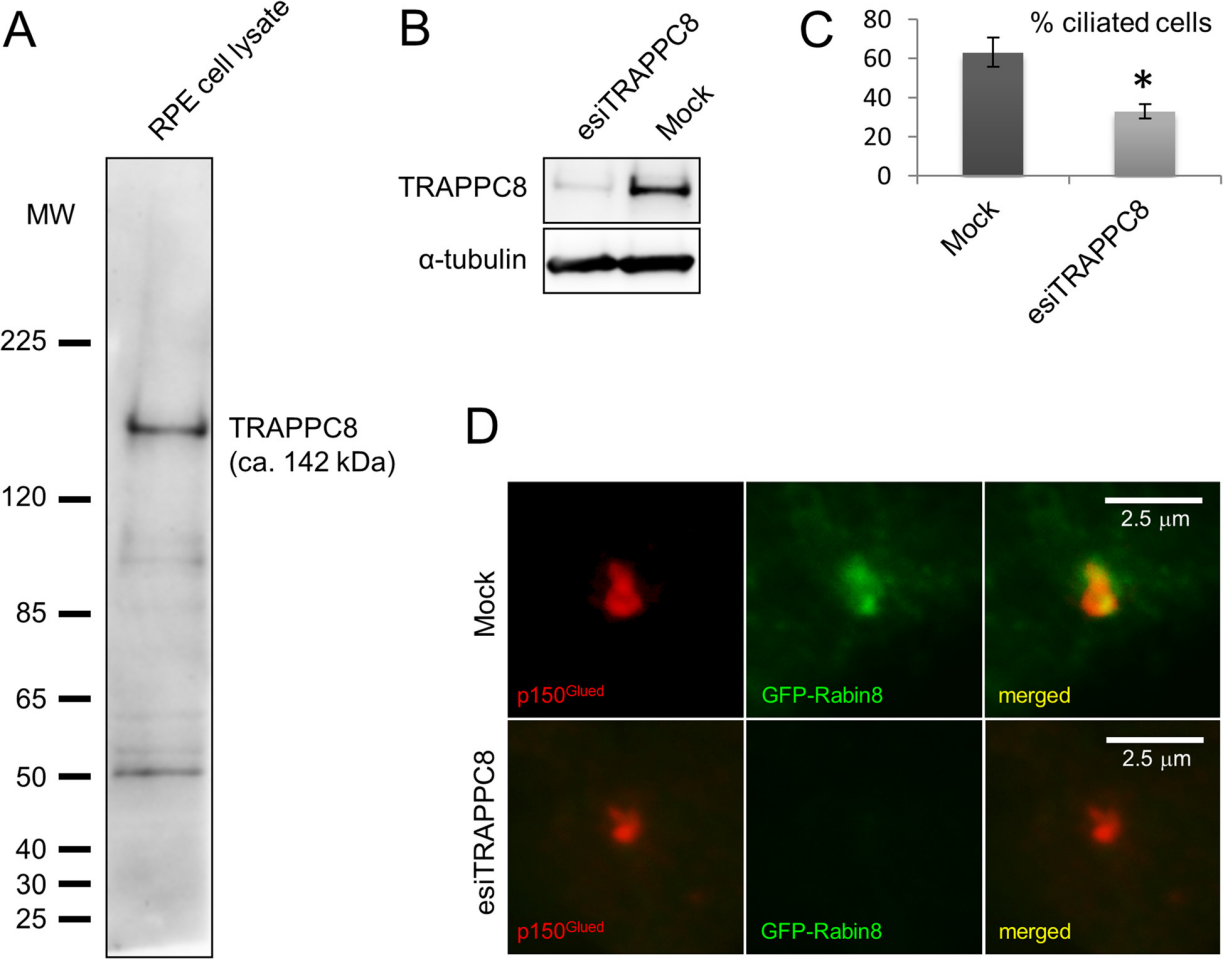
**Identification of a TPR-ASH domain structure in TRAPPII complex subunits. (A)** Domain architecture of known and putative TRAPPII subunits, represented by human TRAPPC8, -9, -10, -11, and -13. Reciprocal HHpred searches identified the regions containing ASH domains, as shown. TRAPPC8 (region 912-1032; local probability 96.98, E = 0.0038), TRAPPC9 (region 725-795; local probability 97.20, E = 0.002), TRAPPC11 (region 748-1133; local probability 96.69, E = 0.012), TRAPPC13 (region 71-150; probability = 97.21, E = 0.0013). The TRAPPC10 C-terminus is more distantly related to the ASH domain. Three iterative HMM-HMM searches of TRAPPC10 region 1072-1158 (local search mode) produced a match to PapD with probability 91.01 and E = 0.85. TPR repeat regions were identified by HHpred and TPRpred (see main text). A minimum of two TPR repeats was used as an exclusion criterium. Human TRAPPC10 was only shown to contain TPR repeats in HHpred. **(B)** Multiple sequence alignment of ASH domains identified previously [27] and here. Sequences were aligned using MAFFT (http://myhits.isb-sib.ch/cgi-bin/mafft) here only representing the partial ASH regions surrounding the conserved asparagine (N). The secondary structure, predicted by HHpred, is shown above the alignment (arrows indicating β-sheets) and the derived consensus amino acids of the alignment are shown below. Consensus abbreviations were obtained in Jalview (http://www.jalview.org/): hydrophobic (h, blue), polar (light green), acidic (violet), glycine (brown), proline (brown green), and asparagine (dark green). For simplicity, only representative proteins found in the first iterative HHpred search with Trs120 against human PFAM profiles are aligned. The representative sequences include: *Schizosaccharomyces pombe* (Sp) Trs120, human (Hs) TRAPPC8, -9, -11, and -13, as well as human DLEC1, Hydin, CCDC108, Cxorf22, RW1, and the PFAM entry of bacterial PapD (as displayed by HHpred). For a graphical output showing results of the initial HHpred search with human DLEC1 (residues 769-980) as search query, see Additional file 1: Figure S1.

**Figure 2:**
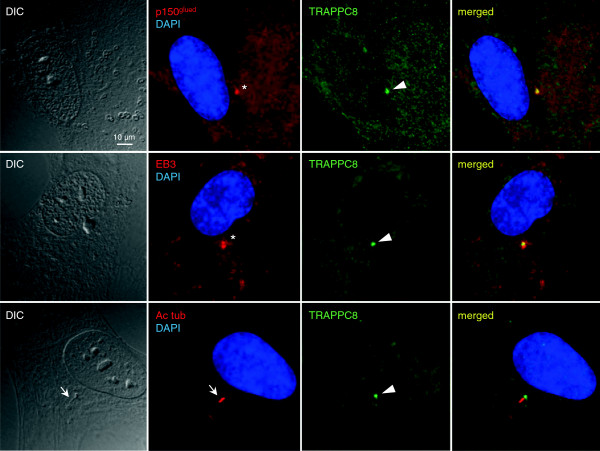
**Predicted 3D structure of the TRAPPII ASH domain. (A)** Known structure of a Hydin ASH domain (PDB (http://www.pdb.org) entry 2E6J). **(B)** Model of the TRAPPC9 ASH structure constructed using Modeller (http://toolkit.tuebingen.mpg.de/modeller) based on the structure of the OCRL1 ASH domain (PDB (http://www.pdb.org) entry 3QBT). **(C)** The solved structure of the bacterial PapD protein is shown for comparison (PDB (http://www.pdb.org) entry 2WMP). **(D)** The OCRL1 ASH structural coordinates were obtained at PDB.org (3QBT).

Encouraged by the above findings, and to uncover the full repertoire of Trs120/TRAPPC9 homologs bearing ASH domains in humans and yeast, we decided to validate the occurrence of this domain in each of the known or putative TRAPPII-specific subunits. In each instance, the minimal C-terminal portion displaying sequence homology to ASH was retrieved and used as a query in HHpred (http://toolkit.tuebingen.mpg.de/hhpred). Remarkably, we were able to show the presence of one or two ASH domains in three of the subunits of yeast TRAPPII (Trs65, -120, and -130) and their human counterparts TRAPPC9, -10, -11, and -13 as well as TRAPPC8 (Figure 1). These searches yielded significant local E values in the range of 10^-2^-10^-3^ with the exception of TRAPPC10 showing more remote similarity to the ASH (probability 91.01 and E = 0.85). Collectively, these searches show that almost all the known and putative TRAPPII-specific components exhibit genuine homology to the ASH/MSP domain (Figure 1). Further, using the Modeller server (http://toolkit.tuebingen.mpg.de/modeller) we could predict a tertiary structure model of each identified ASH domain that was compatible with the solved 3D structure of the human OCRL1 ASH domain [26] (Figure 2 and data not shown). We also confirmed the human OCRL1 ASH region as the best, and statistically most significant, match by mining the Protein Data Bank (PDB) [46] for similar 3D structures, thus reinforcing our predictions of the existence of this domain in TRAPPC8, -9, -10, -11, and -13.

### Identification of TPR repeats in the N-terminus of known and putative TRAPPII components

Interestingly, Trs120 based profile-profile searches also revealed significant similarities of its N-terminus to the N-termini of other TRAPPII components as well as to human Rapsyn (probability 97.75, E = 0.0096) that consist of short tracts of tetratricopeptide (TPR) repeats, indicating that Trs120 bears TPR repeats in its N-terminus. To further examine the TRAPPII complex subunits for the co-occurrence of TPR repeat conformations, the N-terminal portion of each polypeptide sequence was probed for the presence of high α-helical content coinciding with TPR repeat propensity. With the exception of TRAPPC13, all human and yeast TRAPPII subunits were predicted to harbor amino termini containing long α-helical matrices exhibiting high levels of helicity (>50%) and high TPR repeat propensity, as judged by secondary degrees structure prediction algorithms at the HNN server [47] and searches with the TPRpred server [48]. These results suggest that TRAPPC8, -9, -10, and -11 consist of an α-solenoid bearing stretches of multiple TPR repeats followed by one or two ASH domains in the C-terminus (Figure 1).

### The ASH domains of TRAPPC10 and TRAPPC11 target to the centrosome and affect microtubule organization

**Figure 3:**
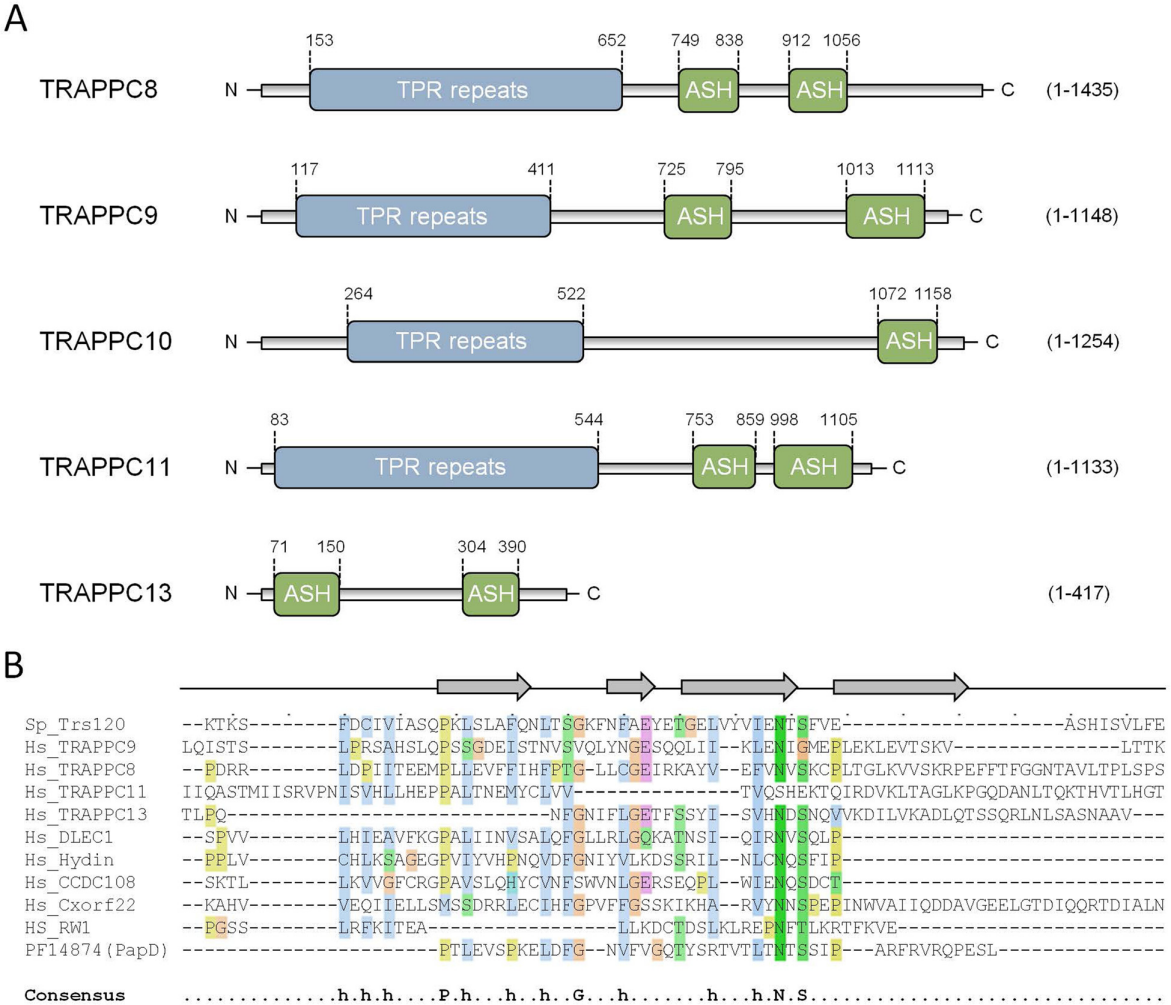
**The TRAPPC10 and TRAPPC11 ASH domains target to the centrosome and affect microtubule organization.** RPE cells expressing Myc-TRAPPC10-ASH or Myc-TRAPPC11-ASH were serum-starved for 24 h, fixed with methanol **(A)** or PFA **(B, C)** and stained with rabbit monoclonal antibody against Myc and mouse monoclonal antibody against α-tubulin (tub), acetylated tubulin (Ac tub) or p150^Glued^, as indicated. DNA was stained with DAPI. Note that the Myc-ASH fusion proteins localize to the centrosome (arrowheads) and basal body (asterisk), and cause bundling of microtubules (open arrow). An example of a primary cilium, stained with Ac tub antibody, is indicated with a closed arrow. Punctate staining near the nucleus can be seen in some cells expressing Myc-ASH fusion proteins (green staining in panel **A)**. Insets show enlargement of the centrosome area.

Since the ASH domain seems to be confined to proteins that localize to the centrosome, Golgi, and the cilium [27], and to validate our bioinformatics results, we analyzed the subcellular localization of two of the identified ASH domains, the ASH domain of TRAPPC10 and the second ASH domain of TRAPPC11 (Figure 1). To this end, plasmids coding for Myc-tagged versions of these two domains were expressed in RPE cells, the cells subjected to 24 h of serum starvation to induce ciliogenesis, and analyzed by immunofluorescence microscopy using rabbit antibodies specific for Myc as well as mouse antibodies against α-tubulin, acetylated-tubulin, or p150^Glued^ to mark the microtubule cytoskeleton, cilia, and centrosome, respectively. As shown in Figure 3, both Myc-ASH domain fusion proteins localized at the centrosome as well as to specific punctae overlapping the nucleus. Moreover, cells overexpressing the Myc-ASH fusion proteins frequently displayed bundles of microtubules surrounding the nucleus (Figure 3A, B), indicating that overexpression of the ASH domain perturbs microtubule dynamics/organization. We conclude that at least two of the identified ASH domains can target to the centrosome.

### Localization of endogenous TRAPPC8 to the centrosome/basal body

**Figure 4:**
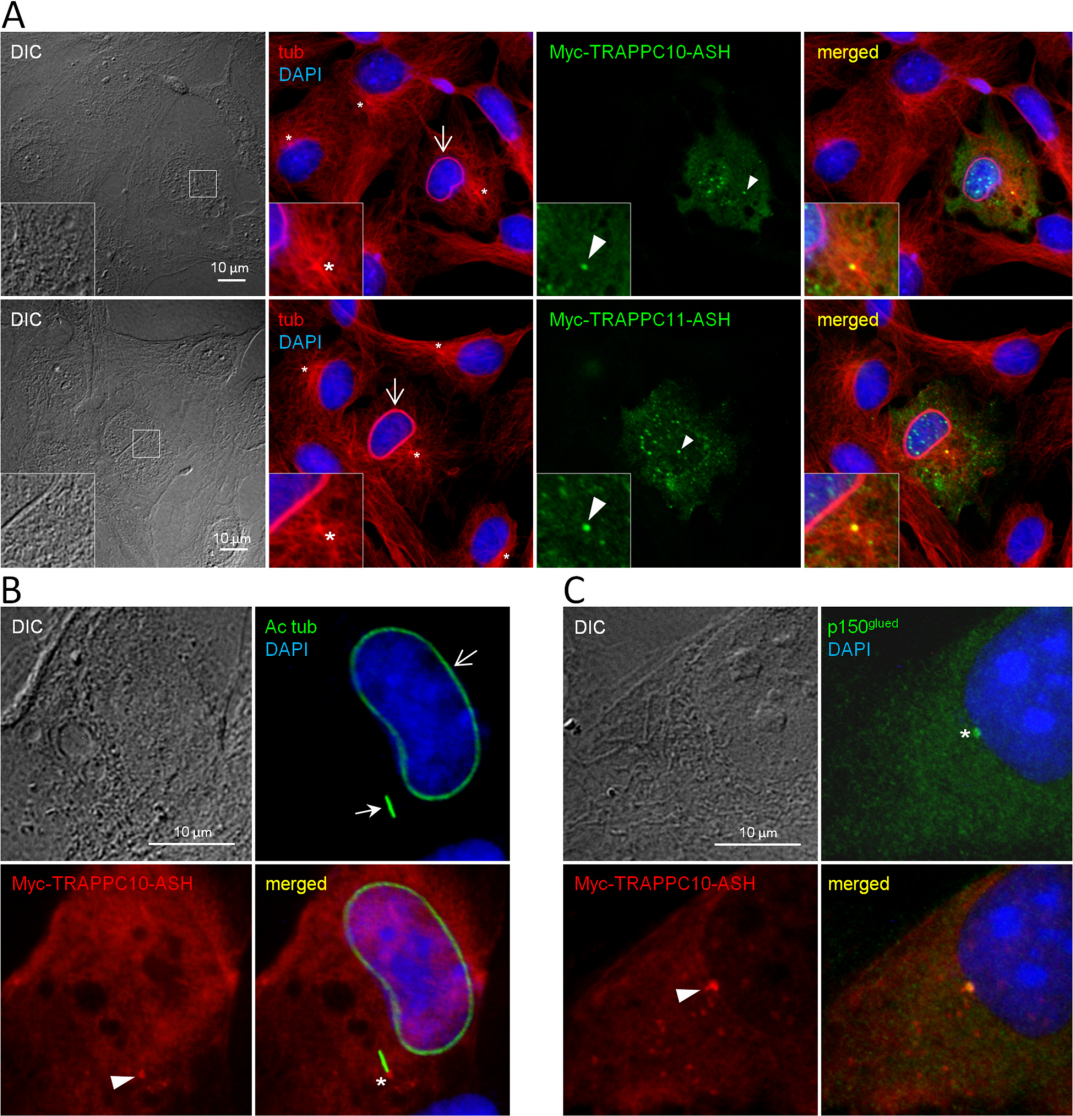
**Western blot analysis and esiRNA-mediated depletion of TRAPPC8. (A)** Western blot analysis of whole cell lysate from RPE cells, probed with rabbit polyclonal TRAPPC8 antibody. Molecular mass markers are shown in kDa to the left. **(B)** Western blot analysis of lysates from RPE cells treated with TRAPPC8-specific esiRNA or mock-transfected control cells. Blots were probed with antibodies specific for TRAPPC8 or α-tubulin (loading control). **(C)** Quantification of cilia in RPE cells depleted for TRAPPC8 using TRAPPC8-specific esiRNA. The cells were fixed with PFA and stained with acetylated tubulin antibody for visualization of cilia. Three independent experiments were conducted with 100 cells counted per condition per experiment. *P* value (*) = 0.0227 using unpaired *t* test. **(D)** Selected immunofluorescence micrographs of GFP-Rabin8 expressing mock-transfected control cells or cells depleted for TRAPPC8. Cells were first treated with mock or TRAPPC8-specific esiRNA and then transfected with GFP-Rabin8 plasmid. Following serum starvation for 1 h, cells were fixed with PFA and stained with antibody against p150^Glued^ to mark the centrosome (red). In Mock-transfected control cells, 92% of GFP-Rabin8 expressing cells showed GFP-Rabin8 at the centrosome whereas only 60% of the GFP-Rabin8 expressing TRAPPC8-depleted cells showed centrosomal GFP-Rabin8 localization (50 cells analyzed per condition).

**Figure 5:**
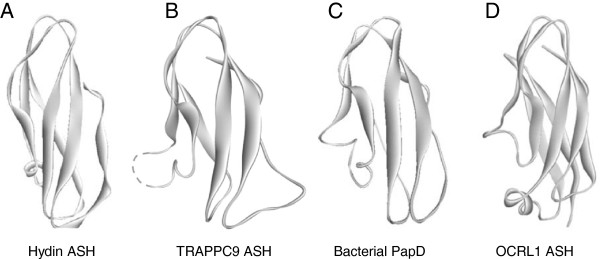
**Endogenous TRAPPC8 localizes to the centrosome/basal body.** RPE cells were serum-starved for 24 h, fixed with methanol (upper two panels) or PFA (lower panel) and stained with rabbit polyclonal antibody against TRAPPC8 (green) and mouse monoclonal antibody against p150^Glued^, rat monoclonal antibody against EB3 or mouse monoclonal antibody against acetylated tubulin (Ac tub), as indicated (red). DNA was stained with DAPI. Arrowheads and asterisks point to the centrosomes/basal bodies. Closed arrow indicates a primary cilium.

To further investigate the link between the ASH domain and centrosome/basal body localization, we next investigated the subcellular localization of endogenous TRAPPC8 in cultured RPE cells. TRAPPC8 has not previously been shown to localize to centrosomes and cilia; commercially available TRAPPC8 antibody recognized a prominent band of appropriate size in western blot analysis of RPE cell lysate that was reduced in intensity upon treatment of cells with TRAPPC8-specific siRNA (Figure 4A, B). Interestingly, immunofluorescence microscopy analysis of serum-starved RPE cells with the TRAPPC8 antibody showed prominent staining of the centrosome/basal body, as revealed by co-staining with antibodies against p150^Glued^, EB3, or acetylated tubulin (Figure 5). Thus TRAPPC8 localizes to the centrosome/basal body. We also used similar approaches to investigate whether TRAPPC11 and TRAPPC13 localize to the basal body/centrosome, but immunofluorescence microscopy analyses with commercially available antibodies against these two proteins were unsuccessful, and hence their subcellular localization could not be addressed. To test if TRAPPC8 is involved in ciliogenesis, as reported previously for TRAPPC3, TRAPPC9, and TRAPPC10 [14], we depleted TRAPPC8 from RPE cells using esiRNA (Figure 4B), subjected cells to serum depletion for 24 h to induce ciliogenesis, and analyzed ciliation frequency by immunofluorescence microscopy with acetylated tubulin antibody. Interestingly, this analysis revealed a significant reduction in ciliation frequency of TRAPPC8-depleted cells compared to mock-transfected control cells (approximately 33% and 63% ciliated cells, respectively; Figure 4C). To investigate if the observed reduction in ciliation frequency of TRAPPC8-depleted cells was linked to defective targeting of Rabin8 to the centrosome, as reported previously for TRAPPC3, TRAPPC9, and TRAPPC10 [14], we expressed GFP-Rabin8 [14] in RPE cells depleted for TRAPPC8 and in mock-transfected control cells. Interestingly, immunofluorescence microscopy of these cells using p150^Glued^ antibody as centrosome marker revealed that TRAPPC8 depleted cells were impaired in their ability to recruit GFP-Rabin8 to the centrosome (Figure 4D). Thus TRAPPC8 localizes to the centrosome/basal body and appears to be required for ciliogenesis, likely via recruitment of Rabin8 to the centrosome. Further experiments will be required to determine whether TRAPPC8 functions together with or separately from the TRAPPII complex in this process.

## Discussion

Activation of the Rab11-Rabin8-Rab8 axis relies on the highly conserved Golgi TRAPPII subunits TRAPPC9 and TRAPPC10, which associate and co-localize with Rabin8 to ensure its targeting to the centrosome and proper biogenesis of the ciliary membrane [14]. Although poorly defined in mammals, yeast data have revealed a division of labor between the two analogous TRAPPI and TRAPPII complexes [17]. Yeast TRAPPI consists of seven subunits (Trs20, Trs23, Trs31, Trs33, Bet3a, Bet3b, Bet5) and the TRAPPII complex includes four additional proteins, Trs65, Trs120, Trs130, and Tca17, which comprise the putative yeast orthologs of mammalian TRAPPC13, TRAPPC9, TRAPPC10/11, and TRAPPC2L, respectively [17-19]. TRAPPI mediates ER to early Golgi trafficking whereas the TRAPPII complex performs late Golgi vesicle tethering for a diverse group of membrane proteins [17]. Despite a wealth of functional insight, and although the overall architecture of the TRAPPII complex has been determined [49], the molecular structure of individual TRAPPII complex subunits and the biochemical basis for how they recognize their targets remain unknown.

**Figure 6:**
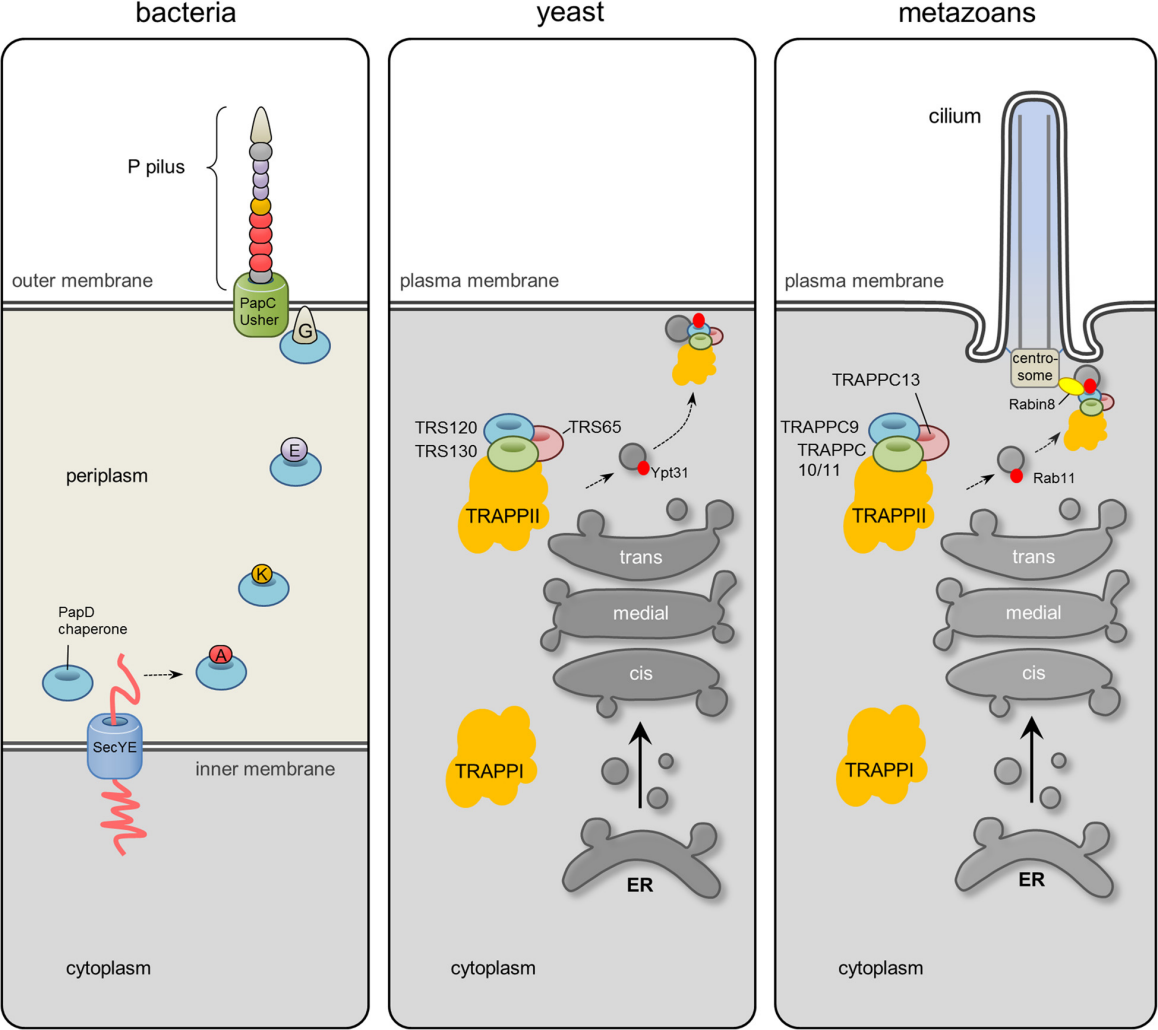
**The TRAPPII complex may have evolved from bacterial PapD chaperone of the usher pili assembly pathway.** Cartoon showing the bacterial and eukaryotic trafficking pathways guided by the PapD and TRAPPII complex, respectively. In bacteria, PapD functions as a chaperone that restrains and tethers the polypeptides to the PapC usher pore complex as they convey across the periplasm between the inner- and outer membrane [35]. In eukaryotes, the TRAPPII particle with its distinct Trs120, Trs130, and Trs65 components (yeast), as compared to the TRAPPI complex, targets proteins in late Golgi trafficking to the outer membrane. In metazoans a similar function of the TRAPPII complex is believed to traffic vesicular cargo to the plasma membrane and cilium [17]. TRAPPC8 may also be involved in Rabin8 centrosome targeting, but it is unclear whether TRAPPC8 functions together with or separately from the TRAPPII complex in this process.

We here shed the first light on the domain organization of conserved TRAPPII complex-specific subunits in yeast their human orthologs with implications for the ciliary targeting and evolution of this protein complex. Based on profile-to-profile searches and structural threading we propose that the TRAPPII-specific subunits are paralogous entities that bear conserved domain arrangements consisting of amino terminal arrays of TPR repeats followed by a C-terminal ASH module (except for TRAPPC13, which does not appear to contain an amino terminal TPR repeat region). Among the subunits that adopt such a TPR-ASH bipartite arrangement, we find the yeast TRAPPII Trs120, Trs130, and Trs65 subunits as well as human TRAPPC8, 9, 10, and 11. In addition, we find that these TRAPP components, as with the MSP modules of VAPB [28], show remote homology to the bacterial periplasmic trafficking chaperone PapD of the usher pili assembly machinery [35] (Figures 1 and 2, and Additional file 1: Figure S1). We therefore propose an ancient relationship between the gram-negative bacterial secretory SecY-dependent pili assembly pathway and the eukaryotic TRAPPII-facilitated vesicular trafficking pathway from the late Golgi to the membrane of the primary cilium (Figure 6).

Our immunofluorescence microscopy analysis showing that heterologously expressed TRAPPC10-ASH and TRAPPC11-ASH localize to the centrosome and affect microtubule organization (Figure 3), as well as our observation that endogenous TRAPPC8 localizes to the centrosome (Figure 5) and is required for ciliogenesis (Figure 4), strongly suggests that the ASH domain constitutes a centrosome-targeting module. How might the ASH domain of TRAPP components confer targeting to the centrosome? One possibility is that the ASH domain directly binds microtubules, since the *Drosophila* ASPM homolog abnormal spindle, asp, binds microtubules within a 512 amino acid region that contains the ASH domain [50]. Alternatively, it is possible that the TRAPP ASH domain interacts with centrosome-specific Rab GTPases such as Rab8. Structural studies of the ASH domain-containing OCRL1 protein showed that this domain directly interacts with Rab8a [26], and TRAPPC9 and TRAPPC10 were previously shown to interact with Rabin8 [14], a GTP exchange factor for Rab8 [15]. Finally, a polypeptide comprising the last 211 amino acid residues of TRAPPC9, which includes all of the second ASH domain (Figure 1A), was shown to interact directly with the NIK and IKKβ-binding protein NIB [51], indicating that the TRAPP ASH domain could also be mediating such interactions. Clearly, more studies are needed to understand how the ASH domain engages in interactions with different binding partners. Such studies will be facilitated by the results of our bioinformatics analysis presented here.

Even though more studies are needed to identify the mechanism by which the ASH domain targets to the centrosome/basal body, several reports indicate that the ASH domains identified here for TRAPPII components are functionally important. For example, a recent report identified a splice site mutation before exon 18 in *TRAPPC9,* leading to formation of a truncated TRAPPC9 polypeptide ending at amino acid 967, in two patients with a Prader-Willi-like phenotype [52], signifying that the C-terminal region of TRAPPC9 containing the second ASH domain (Figure 1A) is functionally important. Similarly, a mutation in *TRAPPC11* leading to a single amino acid change in the region between the two predicted ASH domains (pGly980Arg; Figure 1A) was identified in patients with myopathy, infantile hyperkinetic movements, ataxia, and intellectual ability [53], demonstrating the functional importance of this region.

The identification of ASH/MSP domains in the TRAPPII subunits underpins their previously proposed function in ciliary membrane biogenesis [14] at the molecular level, and corroborates the idea that the ASH domain is associated with cilia-related functions [27]. The presence of an amino terminal α-helical TPR repeat region is also a hallmark of numerous ciliary proteins [54], and hence the presence of such a TPR repeat region in the N-terminus of most TRAPPII subunits (Figure 1) is in line with their ciliary function. As with the ASH domain, the TPR repeat region also seems to be functionally important because mutation leading to deletion of residues 372-429 of TRAPPC11 were shown to impair post-Golgi trafficking and to cause myopathy, infantile hyperkinetic movements, ataxia and intellectual disability in patients [53].

Although we found TRAPPC8 to be localized to the centrosome/basal body (Figure 5) and to be required for ciliogenesis and centrosomal targeting of GFP-Rabin 8 (Figure 4) TRAPPC8 has not been reported to be part of the TRAPPII complex (see Background). Thus it remains to be determined if the observed effects of TRAPPC8 depletion in ciliogenesis involve interaction with TRAPPII components such as TRAPPC9 and TRAPPC10 [14]. In yeast TRAPPC8 (Trs85) functions in autophagy [17]. Interestingly, two recent studies indicated a functional link between primary cilia and autophagy [55,56], suggesting that TRAPPC8 function at the centrosome/basal body could also be linked to autophagy. In future studies it will be interesting to investigate this further and to investigate possible cilia-related function for TRAPPC11 and TRAPPC13.

## Conclusions

Our results suggest that the TRAPP subunits TRAPPC8, -9, -10, and -11 subunits as well as their yeast counterparts exhibit a domain structure consisting of an N-terminal α-solenoid with TPR repeats followed by a C-terminus harboring one or two ASH domains. Immunofluorescence microscopy analysis as well as esiRNA-mediated silencing of TRAPPC8, indicated that the ASH domain is a bona fide centrosome targeting domain, and that TRAPPC8 has a novel function in promoting ciliogenesis. Human genetics studies suggest that both the TPR repeat region and the ASH domain are functionally important, but more work will be required to investigate the detailed structure and function of these domains. Finally, given the structural similarity between TRAPPCII ASH domains and the bacterial trafficking chaperone PapD of the usher pili assembly machinery, our results indicate that the TRAPPCII components may be evolutionarily related to PapD (Figure 6). This would be in line with the autogenous hypothesis of ciliary evolution in which it is suggested that cilia and IFT evolved from coated vesicle transport [57,58].

## Abbreviations

ASH: ASPM, SPD-2, Hydin; BBS: Bardet Biedl syndrome; BLAST: Basic local alignment search tool; DAPI: 4′,6-diamidino-2-phenylindole; DIC: Differential interference contrast; GFP: Green fluorescent protein; HHM: Hidden Markov model; hTERT RPE: Human telomerase-immortalised retinal pigmented epithelial; IFM: Immunofluorescence microscopy; IFT: Intraflagellar transport; MSP: Major sperm protein; MSA: Multiple sequence alignments; OCRL: Oculocerebrorenal syndrome of Lowe protein; PAGE: Polyacrylamide gel electrophoresis; PDB: Protein data bank; PFA: Paraformaldehyde; PSI-BLAST: Position-specific iterative basic local alignment search tool; SDS: Sodium dodecyl sulfate; siRNA: Small interfering RNA; TPR: Tetratricopeptide repeat; TRAPP: Transport protein particle.

## Competing interests

The authors declare that they have no competing interests.

## Authors’ contributions

KBS carried out the bioinformatics analyses and KBS and SKM performed the experiments. KBS, STC, and LBP made the figures. KBS and LBP wrote the paper. All authors read, edited and approved the final manuscript.

## Supplementary Material

Additional file 1: Figure S1 Graphical output showing results of the initial HHpred search with human DLEC1 (residues 769-980) as search query. The amino acid sequence of human DLEC1 (residues 769-980) was used as a search query in HHpred. Profile-to-profile searches were obtained by three iterative PSI-BLAST searches against the PFAM database of HMM profiles. The figure shows a bar graph summarizing the positions and color-coded significances of the database matches with more than 40% probability. The bars are color-coded according to the significance of the hits (For details see http://toolkit.tuebingen.mpg.de/hhpred). From red meaning very significant to blue meaning not significant. Click here for file

## References

[B1] Satir P Pedersen LB Christensen ST The primary cilium at a glance J Cell Sci 2010 123 499 503 10.1242/jcs.050377 20144997 PMC2818190

[B2] Sorokin S Centrioles and the formation of rudimentary cilia by fibroblasts and smooth muscle cells J Cell Biol 1962 15 363 377 10.1083/jcb.15.2.363 13978319 PMC2106144

[B3] Pedersen LB Veland IR Schrøder JM Christensen ST Assembly of primary cilia Dev Dyn 2008 237 1993 2006 10.1002/dvdy.21521 18393310

[B4] Ghossoub R Molla-Herman A Bastin P Benmerah A The ciliary pocket: a once-forgotten membrane domain at the base of cilia Biol Cell 2011 103 131 144 10.1042/BC20100128 21275905

[B5] Clement CA Ajbro KD de Jesus MPR H Koefoed K Vestergaard ML Veland IR Pedersen LB Benmerah A Andersen CY Larsen LA Christensen ST Regulation of TGFβ signaling by endocytosis at the pocket region of the primary cilium Cell Rep 2013 3 1806 1814 10.1016/j.celrep.2013.05.020 23746451

[B6] Hildebrandt F Benzing T Katsanis N Ciliopathies New Engl J Med 2011 364 1533 1543 10.1056/NEJMra1010172 21506742 PMC3640822

[B7] Waters AM Beales PL Ciliopathies: an expanding disease spectrum Pediatr Nephrol 2011 26 1039 1056 10.1007/s00467-010-1731-7 21210154 PMC3098370

[B8] Tobin JL Beales PL Bardet-Biedl syndrome: beyond the cilium Pediatr Nephrol 2007 22 926 936 10.1007/s00467-007-0435-0 17357787 PMC6904379

[B9] Nachury MV Loktev AV Zhang Q Westlake CJ Peranen J Merdes A Slusarski DC Scheller RH Bazan JF Sheffield VC Jackson PK A core complex of BBS proteins cooperates with the GTPase Rab8 to promote ciliary membrane biogenesis Cell 2007 129 1201 1213 10.1016/j.cell.2007.03.053 17574030

[B10] Loktev AV Zhang Q Beck JS Searby CC Scheetz TE Bazan JF Slusarski DC Sheffield VC Jackson PK Nachury MV A BBSome subunit links ciliogenesis, microtubule stability, and acetylation Dev Cell 2008 15 854 865 10.1016/j.devcel.2008.11.001 19081074

[B11] Lechtreck KF Johnson EC Sakai T Cochran D Ballif BA Rush J Pazour GJ Ikebe M Witman GB The Chlamydomonas reinhardtii BBSome is an IFT cargo required for export of specific signaling proteins from flagella J Cell Biol 2009 187 1117 1132 10.1083/jcb.200909183 20038682 PMC2806276

[B12] Jin H White SR Shida T Schulz S Aguiar M Gygi SP Bazan JF Nachury MV The conserved Bardet-Biedl syndrome proteins assemble a coat that traffics membrane proteins to cilia Cell 2010 141 1208 1219 10.1016/j.cell.2010.05.015 20603001 PMC2898735

[B13] Hsiao YC Tuz K Ferland RJ Trafficking in and to the primary cilium Cilia 2012 1 4 10.1186/2046-2530-1-4 23351793 PMC3541539

[B14] Westlake CJ Baye LM Nachury MV Wright KJ Ervin KE Phu L Chalouni C Beck JS Kirkpatrick DS Slusarski DC Sheffield VC Scheller RH Jackson PK Primary cilia membrane assembly is initiated by Rab11 and transport protein particle II (TRAPPII) complex-dependent trafficking of Rabin8 to the centrosome Proc Natl Acad Sci U S A 2011 108 2759 2764 10.1073/pnas.1018823108 21273506 PMC3041065

[B15] Hattula K Furuhjelm J Arffman A Peranen J A Rab8-specific GDP/GTP exchange factor is involved in actin remodeling and polarized membrane transport Mol Biol Cell 2002 13 3268 3280 10.1091/mbc.E02-03-0143 12221131 PMC124888

[B16] Knodler A Feng S Zhang J Zhang X Das A Peranen J Guo W Coordination of Rab8 and Rab11 in primary ciliogenesis Proc Natl Acad Sci U S A 2010 107 6346 6351 10.1073/pnas.1002401107 20308558 PMC2851980

[B17] Yu S Liang Y A trapper keeper for TRAPP, its structures and functions Cell Mol Life Sci 2012 69 3933 3944 10.1007/s00018-012-1024-3 PMC11114727 22669257

[B18] Choi C Davey M Schluter C Pandher P Fang Y Foster LJ Conibear E Organization and assembly of the TRAPPII complex Traffic 2011 12 715 725 10.1111/j.1600-0854.2011.01181.x 21453443

[B19] Scrivens PJ Noueihed B Shahrzad N Hul S Brunet S Sacher M C4orf41 and TTC-15 are mammalian TRAPP components with a role at an early stage in ER-to-Golgi trafficking Mol Biol Cell 2011 22 2083 2093 10.1091/mbc.E10-11-0873 21525244 PMC3113772

[B20] Zong M Wu XG Chan CW Choi MY Chan HC Tanner JA Yu S The adaptor function of TRAPPC2 in mammalian TRAPPs explains TRAPPC2-associated SEDT and TRAPPC9-associated congenital intellectual disability PLoS ONE 2011 6 e23350 10.1371/journal.pone.0023350 21858081 PMC3156116

[B21] Schmidt KN Kuhns S Neuner A Hub B Zentgraf H Pereira G Cep164 mediates vesicular docking to the mother centriole during early steps of ciliogenesis J Cell Biol 2012 199 1083 1101 10.1083/jcb.201202126 23253480 PMC3529528

[B22] Feng S Knodler A Ren J Zhang J Zhang X Hong Y Huang S Peranen J Guo W A Rab8 guanine nucleotide exchange factor-effector interaction network regulates primary ciliogenesis J Biol Chem 2012 287 15602 15609 10.1074/jbc.M111.333245 22433857 PMC3346093

[B23] Ruellas AC Pithon MM Oliveira DD Oliveira AM Lowe syndrome: literature review and case report J Orthod 2008 35 156 160 10.1179/146531207225022599 18809779

[B24] Hyvola N Diao A McKenzie E Skippen A Cockcroft S Lowe M Membrane targeting and activation of the Lowe syndrome protein OCRL1 by rab GTPases EMBO J 2006 25 3750 3761 10.1038/sj.emboj.7601274 16902405 PMC1553191

[B25] Fukuda M Kanno E Ishibashi K Itoh T Large scale screening for novel rab effectors reveals unexpected broad Rab binding specificity Mol Cell Proteomics 2008 7 1031 1042 10.1074/mcp.M700569-MCP200 18256213

[B26] Hou X Hagemann N Schoebel S Blankenfeldt W Goody RS Erdmann KS Itzen A A structural basis for Lowe syndrome caused by mutations in the Rab-binding domain of OCRL1 EMBO J 2011 30 1659 1670 10.1038/emboj.2011.60 21378754 PMC3102282

[B27] Ponting CP A novel domain suggests a ciliary function for ASPM, a brain size determining gene Bioinformatics 2006 22 1031 1035 10.1093/bioinformatics/btl022 16443634

[B28] Tarr DE Scott AL MSP domain proteins Trends Parasitol 2005 21 224 231 10.1016/j.pt.2005.03.009 15837611

[B29] Bork P Holm L Sander C The immunoglobulin fold. Structural classification, sequence patterns and common core J Mol Biol 1994 242 309 320 7932691 10.1006/jmbi.1994.1582

[B30] Coon BG Hernandez V Madhivanan K Mukherjee D Hanna CB Barinaga-Rementeria Ramirez I Lowe M Beales PL Aguilar RC The Lowe syndrome protein OCRL1 is involved in primary cilia assembly Hum Mol Genet 2012 21 1835 1847 10.1093/hmg/ddr615 22228094

[B31] Lechtreck KF Witman GB Chlamydomonas reinhardtii hydin is a central pair protein required for flagellar motility J Cell Biol 2007 176 473 482 10.1083/jcb.200611115 17296796 PMC2063982

[B32] Dawe HR Shaw MK Farr H Gull K The hydrocephalus inducing gene product, Hydin, positions axonemal central pair microtubules BMC Biol 2007 5 33 10.1186/1741-7007-5-33 17683645 PMC2048497

[B33] Lechtreck KF Delmotte P Robinson ML Sanderson MJ Witman GB Mutations in Hydin impair ciliary motility in mice J Cell Biol 2008 180 633 643 10.1083/jcb.200710162 18250199 PMC2234243

[B34] Mahmood S Ahmad W Hassan MJ Autosomal recessive primary microcephaly (MCPH): clinical manifestations, genetic heterogeneity and mutation continuum Orphanet J Rare Dis 2011 6 39 10.1186/1750-1172-6-39 21668957 PMC3123551

[B35] Waksman G Hultgren SJ Structural biology of the chaperone-usher pathway of pilus biogenesis Nat Rev Microbiol 2009 7 765 774 10.1038/nrmicro2220 19820722 PMC3790644

[B36] HHpred Homology detection & structure prediction by HMM-HMM comparisonhttp://toolkit.tuebingen.mpg.de/hhpred24929088

[B37] Soding J Biegert A Lupas AN The HHpred interactive server for protein homology detection and structure prediction Nucleic Acids Res 2005 33 W244 W248 10.1093/nar/gki408 15980461 PMC1160169

[B38] MAFFT MAFFThttp://myhits.isb-sib.ch/cgi-bin/mafft

[B39] Katoh K Misawa K Kuma K Miyata T MAFFT: a novel method for rapid multiple sequence alignment based on fast Fourier transform Nucleic Acids Res 2002 30 3059 3066 10.1093/nar/gkf436 12136088 PMC135756

[B40] Jalview Jalviewhttp://www.jalview.org/

[B41] Modeller Modellerhttp://toolkit.tuebingen.mpg.de/modeller

[B42] Sali A Potterton L Yuan F van Vlijmen H Karplus M Evaluation of comparative protein modeling by MODELLER Proteins 1995 23 318 326 10.1002/prot.340230306 8710825

[B43] Schrøder JM Larsen J Komarova Y Akhmanova A Thorsteinsson RI Grigoriev I Manguso R Christensen ST Pedersen SF Geimer S Pedersen LB EB1 and EB3 promote cilia biogenesis by several centrosome-related mechanisms J Cell Sci 2011 124 2539 2551 10.1242/jcs.085852 21768326 PMC3138699

[B44] Schrøder JM Schneider L Christensen ST Pedersen LB EB1 is required for primary cilia assembly in fibroblasts Curr Biol 2007 17 1134 1139 10.1016/j.cub.2007.05.055 17600711

[B45] Punta M Coggill PC Eberhardt RY Mistry J Tate J Boursnell C Pang N Forslund K Ceric G Clements J Heger A Holm L Sonnhammer EL Eddy SR Bateman A Finn RD The Pfam protein families database Nucleic Acids Res 2012 40 D290 D301 10.1093/nar/gkr1065 22127870 PMC3245129

[B46] PDB Protein Data Bankhttp://www.pdb.org

[B47] HNN HNN secondary structure prediction method http://npsa-pbil.ibcp.fr/cgi-bin/npsa_automat.pl?page=/NPSA/npsa_hnn.html

[B48] TPRpred TPRpredhttp://toolkit.tuebingen.mpg.de/tprpred

[B49] Yip CK Berscheminski J Walz T Molecular architecture of the TRAPPII complex and implications for vesicle tethering Nat Struct Mol Biol 2010 17 1298 1304 10.1038/nsmb.1914 20972447 PMC2988884

[B50] Saunders RD Avides MC Howard T Gonzalez C Glover DM The Drosophila gene abnormal spindle encodes a novel microtubule-associated protein that associates with the polar regions of the mitotic spindle J Cell Biol 1997 137 881 890 10.1083/jcb.137.4.881 9151690 PMC2139842

[B51] Hu WH Pendergast JS Mo XM Brambilla R Bracchi-Ricard V Li F Walters WM Blits B He L Schaal SM Bethea JR NIBP, a novel NIK and IKK(beta)-binding protein that enhances NF-(kappa)B activation J Biol Chem 2005 280 29233 29241 10.1074/jbc.M501670200 15951441 PMC3707486

[B52] Marangi G Leuzzi V Manti F Lattante S Orteschi D Pecile V Neri G Zollino M TRAPPC9-related autosomal recessive intellectual disability: report of a new mutation and clinical phenotype Eur J Hum Genet 2013 21 229 232 10.1038/ejhg.2012.79 22549410 PMC3548258

[B53] Bogershausen N Shahrzad N Chong JX von Kleist-Retzow JC Stanga D Li Y Bernier FP Loucks CM Wirth R Puffenberger EG Hegele RA Schreml J Lapointe G Keupp K Brett CL Anderson R Hahn A Innes AM Suchowersky O Mets MB Nurnberg G McLeod DR Thiele H Waggoner D Altmuller J Boycott KM Schoser B Nurnberg P Ober C Heller R Recessive TRAPPC11 mutations cause a disease spectrum of limb girdle muscular dystrophy and myopathy with movement disorder and intellectual disability Am J Hum Genet 2013 93 181 190 10.1016/j.ajhg.2013.05.028 23830518 PMC3710757

[B54] Taschner M Bhogaraju S Lorentzen E Architecture and function of IFT complex proteins in ciliogenesis Differentiation 2012 83 S12 S22 10.1016/j.diff.2011.11.001 22118932 PMC3977345

[B55] Pampliega O Orhon I Patel B Sridhar S Diaz-Carretero A Beau I Codogno P Satir BH Satir P Cuervo AM Functional interaction between autophagy and ciliogenesis Nature 2013 502 194 200 10.1038/nature12639 24089209 PMC3896125

[B56] Tang Z Lin MG Stowe TR Chen S Zhu M Stearns T Franco B Zhong Q Autophagy promotes primary ciliogenesis by removing OFD1 from centriolar satellites Nature 2013 502 254 257 10.1038/nature12606 24089205 PMC4075283

[B57] Jekely G Arendt D Evolution of intraflagellar transport from coated vesicles and autogenous origin of the eukaryotic cilium Bioessays 2006 28 191 198 10.1002/bies.20369 16435301

[B58] Satir P Mitchell DR Jekely G How did the cilium evolve? Curr Top Dev Biol 2008 85 63 82 19147002 10.1016/S0070-2153(08)00803-X

