# Supplementary figures and images for: Identification of conserved, centrosome-targeting ASH domains in TRAPPII complex subunits and TRAPPC8
Source: Cilia. 2014 Jun 18;3:6. doi: 10.1186/2046-2530-3-6 (PMC4094338; doi:10.1186/2046-2530-3-6)

Figure S1

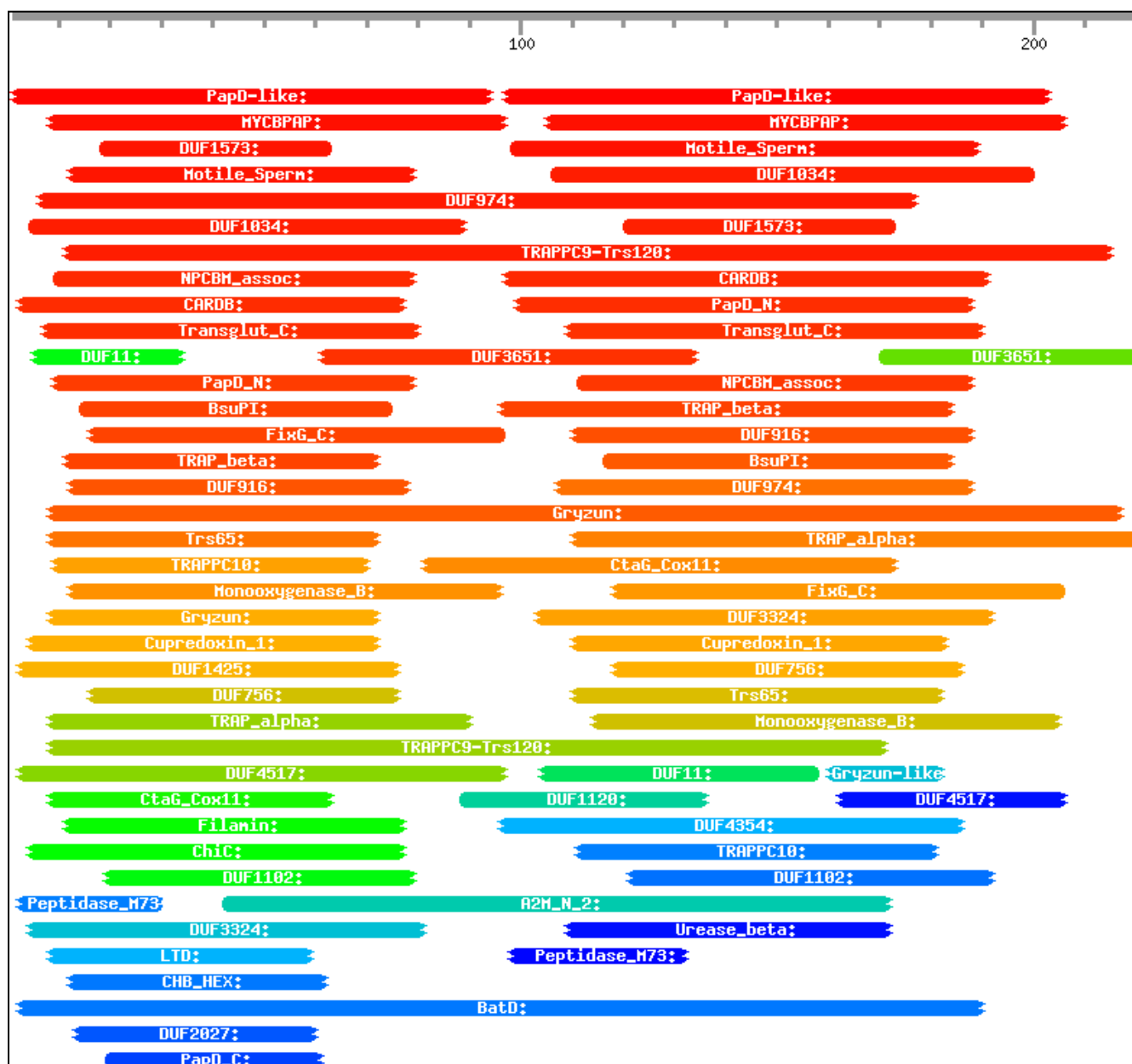

Supplement: Additional file 1: Figure S1 — Graphical output showing results of the initial HHpred search with human DLEC1 (residues 769-980) as search query. The amino acid sequence of human DLEC1 (residues 769-980) was used as a search query in HHpred. Profile-to-profile searches were obtained by three iterative PSI-BLAST searches against the PFAM database of HMM profiles. The figure shows a bar graph summarizing the positions and color-coded significances of the database matches with more than 40% probability. The bars are color-coded according to the significance of the hits (For details see http://toolkit.tuebingen.mpg.de/hhpred). From red meaning very significant to blue meaning not significant. [file 2046-2530-3-6-S1.pdf]
